# Borderline pulmonary pressures in scleroderma - a ‘pre-pulmonary arterial hypertension’ condition?

**DOI:** 10.1186/s13075-015-0649-7

**Published:** 2015-05-18

**Authors:** Gabor Kovacs, Horst Olschewski

**Affiliations:** Medical University of Graz, Graz, 8036 Austria; Ludwig Boltzmann Institute for Lung Vascular Research, Graz, 8010 Austria

## Abstract

Patients with systemic sclerosis may develop borderline pulmonary arterial pressure. The clinical relevance of this condition is not always clear. Reported data support the evidence that this subgroup may represent an intermediate stage between normal pulmonary arterial pressure and manifest pulmonary arterial hypertension, a serious complication in scleroderma. Recognizing the clinical relevance of borderline pulmonary arterial pressure increase in scleroderma patients, future studies should aim for clear evidence for diagnostic and therapeutic algorithms for this population.

In their recent study, Visovatti and colleagues [1] present a detailed analysis of patients with borderline pulmonary arterial pressure (PAP) as a subgroup analysis of the DETECT study, providing important clinical data for understanding early pulmonary vasculopathy in patients with systemic sclerosis.

In fact, every physician who has observed the dramatic deterioration of patients with pulmonary arterial hypertension (PAH) and successive right ventricular failure would urge for the earlier recognition and therapy of this devastating condition. About 10% of all scleroderma patients may develop PAH [2], which - besides lung fibrosis - represents the most frequent cause of death in this patient population [3]. But can PAH be recognized at an early stage and maybe even prevented?

If we assume that the increase of PAP is a process lasting for a longer period of time, there must be a phase of transition from normal (mean PAP ≤20 mmHg) pulmonary hemodynamic conditions to PAH (mean PAP ≥25 mmHg). Patients in this so-called 'borderline' range may represent the early stage of PAH. Earlier studies found that such patients were more likely to develop pulmonary hypertension than patients with mean PAP ≤20 mmHg, with a hazard ratio of 3.7 [4]. The rate of borderline patients developing PAH was 19% after 3 years and 27% after 5 years. Accordingly, we may argue that borderline PAP is a 'pre-PAH' condition in scleroderma. Of course, borderline elevation of PAP may be caused not only by pulmonary vasculopathy but also by cardiac or pulmonary co-morbidities [5]. In these cases borderline elevation of PAP may be considered as a general prognostic marker [5,6].

The analysis of Visovatti and colleagues [1] includes several clinical (for example, current/past telangiectasis, presence of peripheral edema), laboratory (for example, ACA antibody, NT-proBNP), lung functional (for example, forced vital capacity (percentage predicted)/diffusion capacity for carbon monoxide ratio) and cardiac (for example, tricuspid annular plane systolic excursion) markers that may distinguish scleroderma patients with borderline PAP elevation from those with normal PAP or with manifest PAH. According to this analysis, borderline elevation of PAP in scleroderma patients may represent an intermediate stage in the continuum between normal PAP and manifest PAH.

Among the DETECT population, 15% of all patients presented with borderline PAP hemodynamics. Although this number may be different in the general scleroderma population, due to the strict inclusion and exclusion criteria of the DETECT study [7], the borderline population seems to be a substantial subgroup. Unfortunately, follow-up data of the described patients in comparison with normal PAP and manifest PAH patients have not been provided. Such data might impact the development of clinical algorithms regarding further follow-up and treatment of these patients.

In addition to the borderline elevation of resting PAP, another specific hemodynamic situation in scleroderma patients needs careful interpretation: exercise-induced PAP increase. Earlier studies showed that this may be a frequent condition among scleroderma patients and clinical deterioration and the development of PAH are frequent in this population [2]. In a recent analysis, a strong correlation between resting and exercise PAP values was evident [5], suggesting that patients with borderline hemodynamics and those with a strong PAP increase during exercise may strongly overlap, closing the gap between these two hemodynamic conditions.

The most important question remains open: should targeted PAH therapy be offered to scleroderma patients with borderline PAP or exercise-induced PAP increase? Unfortunately there has been no clinical study investigating patients with borderline PAP so far and only two small studies have selected patients with exercise-induced PAP increase [8,9]. The results of these studies are promising, but need to be confirmed in adequately powered, randomized, prospective trials.

Based on a series of studies indicating borderline hemodynamics has an important role in scleroderma patients with regard to the development of PAH and potentially for early treatment, future studies should aim for clear evidence for diagnostic and therapeutic algorithms for this patient population. This may contribute to a substantial prognostic improvement for patients with scleroderma who develop pulmonary vasculopathy

## Abbreviations

PAH: Pulmonary arterial hypertension
PAP: Pulmonary arterial pressure

## References

[1] Visovatti SH, Distler O, Coghlan J, Denton CP, Grunig E, Bonderman D (2014). Borderline pulmonary arterial pressure in systemic sclerosis patients: a post-hoc analysis of the DETECT study. Arthritis Res Ther..

[2] Condliffe R, Kiely DG, Peacock AJ, Corris PA, Gibbs JS, Vrapi F (2009). Connective tissue disease-associated pulmonary arterial hypertension in the modern treatment era. Am J Respir Crit Care Med..

[3] Tyndall AJ, Bannert B, Vonk M, Airo P, Cozzi F, Carreira PE (2010). Causes and risk factors for death in systemic sclerosis: a study from the EULAR Scleroderma Trials and Research (EUSTAR) database. Ann Rheum Dis..

[4] Valerio CJ, Schreiber BE, Handler CE, Denton CP, Coghlan JG (2013). Borderline mean pulmonary artery pressure in patients with systemic sclerosis: transpulmonary gradient predicts risk of developing pulmonary hypertension. Arthritis Rheum..

[5] Kovacs G, Avian A, Tscherner M, Foris V, Bachmaier G, Olschewski A (2014). Characterization of patients with borderline pulmonary arterial pressure. Chest..

[6] Weitzenblum E, Hirth C, Ducolone A, Mirhom R, Rasaholinjanahary J, Ehrhart M (1981). Prognostic value of pulmonary artery pressure in chronic obstructive pulmonary disease. Thorax..

[7] Coghlan JG, Denton CP, Grunig E, Bonderman D, Distler O, Khanna D (2014). Evidence-based detection of pulmonary arterial hypertension in systemic sclerosis: the DETECT study. Ann Rheum Dis..

[8] Saggar R, Khanna D, Shapiro S, Furst DE, Maranian P, Clements P (2012). Brief report: effect of ambrisentan treatment on exercise-induced pulmonary hypertension in systemic sclerosis: a prospective single-center, open-label pilot study. Arthritis Rheum..

[9] Kovacs G, Maier R, Aberer E, Brodmann M, Graninger W, Kqiku X (2012). Pulmonary arterial hypertension therapy may be safe and effective in patients with systemic sclerosis and borderline pulmonary artery pressure. Arthritis Rheum..

